# Enhancing nutritional and functional properties of rice starch by modification with Matcha extract

**DOI:** 10.1002/fsn3.4087

**Published:** 2024-03-08

**Authors:** Hümeyra Cetin‐Babaoglu, Hümeyra Aydın, Rumeysa Kumas, Sultan Arslan‐Tontul

**Affiliations:** ^1^ Food Engineering Department, Agricultural Faculty Selçuk University Konya Turkey

**Keywords:** resistant starch, starch digestibility, starch inclusion complex, tea polyphenols

## Abstract

The aim of this study is to increase the functionality of rice starch by modifying matcha tea extract and to determine the effect on some physicochemical properties and starch digestibility. According to the data analyzed, treatment with matcha extract was effective in increasing the nutritional value of native rice starch. At the highest level of extract addition, total phenolic and flavonoid content reached 129.54 mg/100 g and 40.16 mg/100 g, respectively, as no phenolic or flavonoid content was detected in control. In addition, the highest DPPH and FRAP values were determined to be 296.62 μmol TE/100 g and 814.89 mg/100 g, respectively, at the highest extract addition level. Treatment with matcha extract significantly reduced the eGI of native rice starch from to 94.61 to 64.63, while resistant starch was increased from 0.90 to 33.43%. According to the physiochemical analysis, there was a positive correlation between the extract ratio and the water‐holding capacity of rice starch due to the high hydrophilic capacity of the phenolic compounds. In addition, the solubility and swelling power of starch were increased by treatment with matcha extract, but high temperatures had a negative effect on these physicochemical properties.

## Graphical abstract

The total phenol and flavonoid content of rice starch were increased by MTE treatment. The eGI level of rice starch was decreased from 94.61 to 64.63. Resistant starch content was 30 times higher in S8 sample than native rice starch.

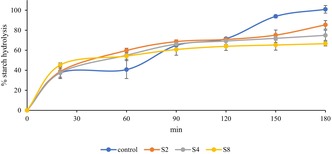

## 1 INTRODUCTION

Starch is a staple dietary carbohydrate source and comprises the main ingredient in most foods. Besides being a key energy supply, starch has low nutritive value in terms of functional compounds. On the other hand, the high glycemic response of starchy foods has a negative side, as the incidence of chronic diseases increases dramatically worldwide. Therefore, recent studies have focused on decreasing glycemic response by delaying glucose release from starch and redesigning the structure of starch to enzyme‐resistant forms. From this point of view, the modification of starch with various bioactive compounds gains importance. However, there is still limited research on the modification of starch with various phytochemicals (Dupuis et al., 2017).

Starch is a macromolecule comprised fractions of amylose and amylopectin. Amylose is in linear form and has much of the making capacity of inclusion complex. The exterior of single left‐handed helix is hydrophilic, but the interior of the helical channel is hydrophobic, thus this structure makes it suitable for hosting guest molecules of the right size (Conde‐Petit et al., 2006). The studies showed that six helical structures of amylose can make complexes with fatty acids, phenolic acids, and flavoring compounds. Starch inclusion complexes are also characterized by the structure of the V‐type amylose complex (Conde‐Petit et al., 2006; Igoumenidis et al., 2018). Most of the research reported that the formed V‐type complex can show effects as dietary fibers and limit starch digestion (Hernández et al., 2022).

In the past decades, considerable research efforts have been devoted to several novel methods for the modification of starch. Among these studies, polyphenol–starch inclusion complex takes an important place in order to decrease glycemic index. Three potential mechanisms of the phenolic starch complex have been reported (Hernández et al., 2022). First of all, phenolics can inhibit the key amylolytic enzymes, thus starch digestion can be limited. In the previous report of Koh et al. (2010), polyphenols, mainly theaflavins and catechins, from different tea infusions were shown to have inhibitory activity on human salivary α‐amylase and mammalian α‐glucosidase. Miao et al. (2015) designed a study to understand the amylase inhibition mechanism of tea extract. The authors discovered that polyphenols adhered with α‐amylase to form a new complex, and as a result, starch digestibility was limited. Additionally, the affinity degree was higher in epicatechin gallate. Second mechanism is that phenolics make inclusion complex, reducing starch accessibility. Lastly, phenolic–starch complex may inhibit glucose transporters (Hernández et al., 2022).

Matcha is the powder of the leaf of *Camellia sinensis*. Numerous papers have documented the significant potential of matcha tea in averting a range of chronic illnesses, including cancer, diabetes, heart disease, obesity, and osteoporosis. These health‐promoting effects of tea are mostly associated with its rich polyphenol content (Koh et al., 2010; Xu et al., 2016). The major phenolic substances present in green tea were reported as epicatechin (59.2%), epigallocatechin gallate (14.6%), and epicatechin gallate (26.2%) (Miao et al., 2015). There has been limited research in the literature to determine the ability of matcha tea extract and starch to form inclusion complexes. Most studies concentrate on the thermal, retrogradation, and pasting properties of starches following the direct addition of the polyphenols, and the limiting of starch digestion and formation of resistant starch have paid little attention. Therefore, the aim of this study is to increase the functionality of rice starch by modification with matcha extract (ME) and determine the effect of some physicochemical properties and starch digestibility.

## 2 MATERIALS AND METHODS

### 2.1 Preparation of matcha extract (ME) and starch complex

Matcha powder, which originated from Japan, was obtained from a global tea shop in Türkiye. To prepare matcha tea, 15 g of powder was suspended in 60‐mL water and stirred at a magnetic stirrer at 70°C for 30 min. After that, the tea suspension was centrifuged at 1000 x *g* for 10 min and the supernatant was filtered through a 0.45 μm filter. After extraction, the total phenolic, flavonoid content, and DPPH radical scavenging activity of the ME were determined to be 54.8 g/kg, 39.1 g/kg, and 121.9 mMol/kg, respectively.

The extract is used to produce rice starch complex. Rice starch was purchased from a starch supplier in Türkiye (Smart Kimya, İzmir, Türkiye). The proximate composition of starch contained 88% total starch, 10.5% moisture, 0.7% total fat, and 0.5% protein. Twenty grams of rice starch was suspended with 300 mL of distilled water and the pH value was adjusted to 6.5 by 0.1 N HCl. For gelatinization, the starch suspension was left in a shaking bath at 90°C for 15 min at 90 rpm. After that, 10% (S2), 20% (S4), and 40% (S8) (v/w of starch) extract were added into the suspension and shaken in a water bath at 90°C for 30 min. The suspension was cooled to room temperature and left for complexation at ambient temperature. After overnight hardening, the starch suspension was filtered, washed with 50% methanol solution, and dried at 50°C for 12 h.

### 2.2 Determination of physicochemical properties of starch complex

The effect of ME on the physicochemical properties of rice starch was determined by following water‐holding capacity (WHC), solubility, and swelling power (SP). To determine the WHC of the complex, starch samples were suspended with distilled water (0.7%, w/v) and left for constant stirring for 1 h. After stirring, the aqueous starch sample was centrifuged at 6350 x *g* for 10 min at room temperature. The upper water phase was removed, and the pellet was weighted (Li et al., 2019).

Solubility and swelling power were determined according to the procedure outlined by Li et al. (2019) with some modifications. Starch samples were suspended with distilled water (0.2%, w/v) and incubated in a shaking water bath at 55°C and 95°C for 1 h. After incubation, samples cooled to room temperature, and aqueous starch samples were centrifuged at 3000 rpm for 20 min. The supernatants were taken and dried at 70°C for 12 h. Finally, the gelatinized wet starch sediment and dried supernatants were weighed. Solubility and swelling power were calculated by Equations 1 and 2:

(1) $$\text{Solubility}\;\left(\%\right)=\frac{\text{Weight of dried supernetant}}{\text{Sample of weight}}\times 100$$

(2) $$\text{Swelling power}\;\left(\%\right)=\frac{\text{Weight of}\;\mathrm{wet}\;\text{sediment}}{\text{Sample of weight}\times \left(100\%-\text{solubility}\%\right)}\times 100$$

### 2.3 Thermal properties

Thermal properties of ME‐treated rice starch samples were performed by Differential Scanning Calorimeter (DSC25; TA Instruments, USA). A 2.5 mg of starch sample was weighed into an aluminum DSC pan and 7.5 μL of distilled water was added. DSC pans were hermetically sealed and left for 24 h at 4°C. The changes in thermal properties were recorded at 25–160°C with 10°C/min heating rates. An empty pan was used as a reference (Zhu et al., 2009).

### 2.4 Determination of total phenolic and flavonoid content of complex

One‐gram grounded starch complex was suspended with 80% methanol (v/v) solution and incubated in a shaking water bath at 45°C for 1 h. After incubation, supernatant was obtained by centrifugation at 3000 rpm for 10 min.

To determine the total phenolic content of the complex, 250‐μL extract, 750‐μL distilled water, and 2.5 mL of 10% Folin reagent (v/v) were added into a tube and left for 5 min in the darkness. At the end of the time, 2‐mL 7.5% NaCO_3_ (w/v) solution was added into the sample tube and left for incubation at 50°C for 5 min. The absorbance of samples was measured at 760 wavelengths by a spectrophotometer. The total phenolic acid content of samples was calculated by a gallic acid standard curve on a dry basis (Škerget et al., 2005).

To determine total flavonoid content, 1‐mL extract, 2.5 mL of distilled water, and 150 μL of 5% NaNO_3_ (w/v) were mixed in a tube and left for 5 min in the darkness. At the end of the time, 300 μL of 10% AlCl_3_ (w/v) solution, 1 mL of 1 N NaOH, and 550‐μL distilled water were added into the sample tube and left for 5 min. The absorbance of samples was measured at 510 wavelengths by a spectrophotometer. The total flavonoid content of samples was calculated by a quercetin standard curve on a dry basis (Eyiz et al., 2020).

### 2.5 Determination of DPPH free‐radical scavenging activity and ferric‐reducing antioxidant power (FRAP)

DPPH and FRAP assays were carried out by extract obtained in Section 2.4 and performed according to our previous studies by Çetin‐Babaoğlu et al. (2021). The standard curve for DPPH was prepared by Trolox (6‐hydroxy‐2,5,7,8‐tetra‐methyl‐chroman‐2‐carboxylic acid), and results were expressed as μM TE/100 g on a dry basis. Additionally, standard curve for FRAP was prepared by iron (II) sulfate heptahydrate, and results were expressed as mg/kg on a dry basis.

### 2.6 In vitro starch digestion and expected glycemic index (eGI)

The in vitro starch hydrolysis of starch complex was determined according to Goñi et al. (1997). Digestion time and hydrolyzed starch were plotted, and a nonlinear starch hydrolysis curve was shaped. To determine the glycemic index, the *C*_∞_ and *k* were calculated from Equation 3, and they were applied to describe the kinetics of starch hydrolysis.

(3) $$C=C_{\infty }\left(1-e^{-\mathrm{kt}}\right)$$

*C* = Concentration of each time
*C*_∞_ = Concentration at equilibrium
*k* = Kinetic constant
*t* = time

The area under the curve (AUC) was calculated from *C*_∞_ and *k* constant (Equation 4).

(4) $$\mathrm{AUC}=C_{\infty }\left(t_{f}-t_{0}\right)-\frac{C_{\infty }}{k}\;\left(1-e^{-k\left(t_{f}-t_{0}\right)}\right)$$

The hydrolysis index (HI) was calculated as the relation between the AUC of MTE‐added starch samples and control (rice starch without extract). Finally, eGI was calculated by Equation 5.

(5) $$\mathrm{eGI}=39.71+0.549\;\left(\mathrm{HI}\right)$$

### 2.7 Statistical analysis

Starch complexation and analysis were conducted as two replicates. After variance analysis of the data, Duncan's multiple range test (*p* < .05) was used to determine the acceptable mean separation. SAS Statistical Software (SAS Institute Inc., Cary, NC, USA) was used for all statistical computations.

## 3 RESULT AND DISCUSSION

### 3.1 Water‐holding capacity (WHC), solubility, and swelling power (SP)

**FIGURE 1:**
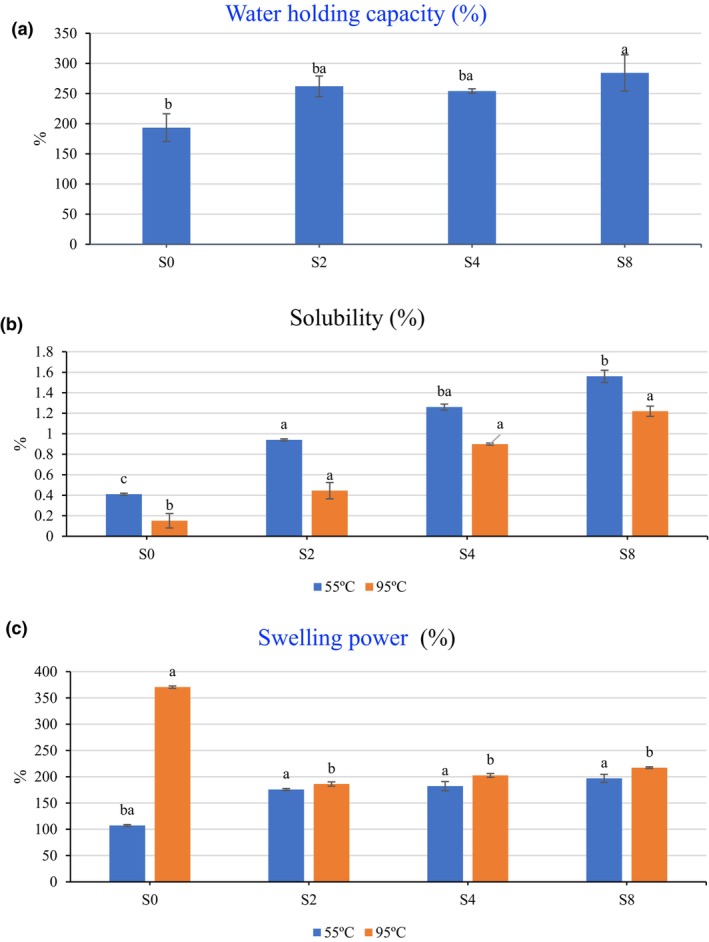
Physicochemical changes in rice starch. S0, untreated with MTE; S2, treated with 10% MTE (v/w of starch); S4, treated with 20% MTE (v/w of starch); S8, treated with 40% MTE (v/w of starch).

The changes in the physicochemical properties of rice starch with the addition of ME are apparent in Figure 1a. According to the results, there was a positive correlation between increasing extract ratio and WHC. The WHC was 193.49% in the control sample and increased to 284.37% in the S8. This result may be attributed to the high hydrophilic capacity of phenolic compounds. By increasing phenolic concentration, the starch complex may bind more water resulting in an increase in WHC. The findings are consistent with Zeng et al. (2020) who determined that the WHC of wheat starch showed an increased trend with the increase of tannic acid concentration. The authors also noted that tannic acid enhanced the water retention capacity and made easier gelatinization by promoting the expansion of starch molecules. Ou et al. (2001) created another perspective on phenolic–starch complex by synthesizing starch ferulic acid esters. It was revealed that the WHC increased significantly in esters and these results suggested that it was beneficial for increasing WHC of intestinal digesta and protecting against constipation.

Solubility is another important physicochemical feature for starch complex since the higher solubility values mean a homogenous mixture of food ingredients and desired food texture. The data in Figure 1b indicated that the addition of ME increased the solubility of starch in tested temperatures of 55°C and 95°C. However, high temperatures had a negative effect on solubility. At 55°C, the solubility index was measured as 0.41% for S0 (control) and it was 1.56% in S8. Additionally, at 95°C, the solubility index was determined as 0.15% for S0, and it was 1.22% for S8. In all samples, solubility was decreased by raising test temperature. Phenolic acids are known as having high hydrogen bond‐making capacity. In this study, phenolics of ME, present in the complex form of rice starch, may lead to being more soluble by making hydrogen bound, and as a result of this solubility index increased. In addition, ME treatment may lead to an increase in the contact surface area of water in starch by gaining a more porous structure. Zheng et al. (2020) observed more pores with caffeic acid treatment of maize starch. Similar results were reported in previous study by Han et al. (2020) who researched the effect of free polyphenols (ferulic acid, gallic acid, and quercetin) on the physicochemical properties of rice starch. It was found that increasing the ratio of phenolic acids enhanced the solubility of starch. The authors attributed these results to the interactions between the hydroxyl groups of phenolic compounds, dissolved in water, change ionic strength and water activity of the aqueous starch solution. Therefore, such a modification encourages soluble starch to dissolve. On the other hand, high temperature may cause more crystalline forms in starch; thus, the solubility decreases.

The degree of interaction between starch chains within the crystalline and amorphous domains of the starch granule can be assessed using swelling power (SP) (Zhao et al., 2019). It is an important rheological feature of all types of starch. It was indicated from Figure 1c that ME addition significantly affected the SP of rice starch. At lower test temperatures, ME promoted SP due to higher rate of amylose leakage; however, controversial result was obtained at the higher test temperature. At 55°C, the SP index was measured as 107.26% in S0 and it was 196.87% in S8. In addition, at 95°C, the SP was determined as 370.51% in S0, and it was 186.19% in S2. The decrease in SP at higher temperatures may be sourced from the formation of hydrogen bonds between phenolic acids and water molecules by hydroxyl and carboxyl groups, which likely restrict water access to amylose and amylopectin (Han et al., 2020). Similar results were reported by Kan et al. (2022) who found that the swelling power significantly increased when wheat starch was complexed with tannic acid.

### 3.2 Thermal characteristics of modified starch

DSC characteristics of ME–rice starch complex are given in Table 1. On the thermogram, two basic endothermic peaks were observed except in native rice starch. The gelatinization temperature (*T*_p_) and thermal energy needed for the gelatinization process of starch (ΔH) were measured in native rice starch at 76.49°C and 2.76 J/g. The detected *T*_p_ and ΔH of the first gelatinization point decreased gradually by ME treatment. In S0, *T*_p_ and ΔH were measured as 70.52°C and 0.28 J/g, and these values were 58.83 and 0.07 J/g for the S8. According to Kan et al. (2022), there were three endothermic transitions with tannic acid–starch complex that occurred at different temperatures. The authors associated the first transition with the melting of crystalline amylopectin structures at 60°C, and the second and third transitions with the formation of the inclusion complex at 130–150°C. The authors also found that the melting point and enthalpy decreased with the addition of tannic acid. A similar result was reported in previous studies of Dupuis et al. (2017) who found that the inclusion of starch with vanillic acid led to decrease gelatinization temperatures and enthalpy, and they attributed these results to changes in gelatinization characteristics of free hydroxyl groups on the vanillic acid. A similar point of view was also revealed by Guo et al. (2019).

**TABLE 1:** Thermal properties of matcha tea extract‐treated rice starch samples.

Sample	First peak		Second peak	
	*T*_p_ (°C)	ΔH (J/g)	*T*_p_ (°C)	ΔH (J/g)
S0	76.49 ± 0.09^a^	2.76 ± 0.65^a^	109.46 ± 1.52^a^	0.57 ± 0.14^a^
S2	70.52 ± 1.99^ba^	0.57 ± 0.01^b^	107.13 ± 0.76^a^	0.46 ± 0.01^a^
S4	63.41 ± 5.36^bac^	0.32 ± 0.01^b^	97.36 ± 2.53^b^	0.15 ± 0.01^b^
S8	54.58 ± 0.76^c^	0.28 ± 0.00^b^	94.75 ± 0.54^b^	0.17 ± 0.04^b^

*Note*: The superscript letters, in the same column, indicate that are significantly different by Duncan's multiple range test (*p <* .05).

Abbreviations: S0, untreated with MTE; S2, treated with 10% MTE (v/w of starch); S4, treated with 20% MTE (v/w of starch); S8, treated with 40% MTE (v/w of starch).

Similar decreasing effects were also obtained at the second gelatinization point. The increase in tea extract significantly decreased the *T*_p_ and ΔH values. This second peak observed in the thermogram was mostly associated with the melting/dissociation of amylose–lipid inclusion complexes (Genkina et al., 2015). The decrease in ΔH value was mostly associated with being less susceptibility to polyphenols interacting with the starch biopolymer chains through hydrogen bonding during gelatinization during reordering (Guo et al., 2019). Moreover, the thermogram data of Lv et al. (2020) revealed that the fortification of wheat starch by tea polyphenols retarded starch retrogradation. It was obvious that changes in the crystalline region and redistribution of water within the complexes contribute to changes in the thermal behavior of starches (Ngo et al., 2022).

### 3.3 Total phenolic and flavonoid content of modified Rice starch

Native starch is the main source of carbohydrate, and most of the daily energy requirement is provided by starch. On the other hand, starch lacks the most bioactive compound. By modification of starch with phenolic‐rich extracts, the poor nutritive value of starch can be improved.

As expected, total phenolic and flavonoid content increased by treatment with ME (Table 2). No phenolic or flavonoid content was detected in S0 (data not shown); however, at the highest extract addition level, total phenolic and flavonoid content reached to 129.54 mg/100 g and 40.16 mg/100 g, respectively. This result indicated that the complexation process was achieved as the content of bioactive compounds increased compared to native starch. As it is known, amylose with helical cavities is to form inclusion compounds with several phenolics on the point of making noncovalent interactions, and thus, the structure and nutritional properties of starch can be modified (Zhu et al., 2009). Zeng et al. (2020) reported that the modification of wheat starch with tannic acid had a positive effect on the nutritional value of starch. Dupuis et al. (2017) also declared that the complexation of potato starch with vanillic acid led to an increase total phenolic content of complexes at various pH ranges.

**TABLE 2:** Antioxidant activity, total phenolic and flavonoid content of matcha tea extract‐treated rice starch samples.

Sample	Total phenolic content (mg/100 g)	Total flavonoid content (mg/100 g)	Antioxidant activity	
			DPPH (μmol TE/100 g)	FRAP (mg/100 g)
S2	33.61 ± 2.33^b^	11.18 ± 0.15^c^	94.31 ± 1.24^c^	251.72 ± 3.86^c^
S4	44.99 ± 0.58^b^	12.81 ± 0.28^b^	119.79 ± 1.82^b^	300.20 ± 5.31^b^
S8	129.54 ± 21.5^a^	40.16 ± 0.28^a^	296.62 ± 2.86^a^	814.89 ± 13.48^a^

*Note*: The superscript letters, in the same column, indicate that are significantly different by Duncan's multiple range test (*p <* .05).

Abbreviations: S2, treated with 10% MTE (v/w of starch); S4, treated with 20% MTE (v/w of starch); S8, treated with 40% MTE (v/w of starch).

### 3.4 Total antioxidant activity of modified Rice starch

DPPH and FRAP values of ME‐treated rice starch complexes are shown in Table 2. Similar to total phenol and flavonoid content data, treatment significantly affected total antioxidant activity of rice starch. The highest DPPH radical scavenging activity was determined S8 sample as 296.62 μmol TE/100 g. It was followed by S4 and S2 samples. Additionally, FRAP value was determined as 814.89 mg/100 g in the S8 sample, and it was followed by 300.20 and 251.72 mg/100 g in S4 and S2 starch samples, respectively. These results showed that ME treatment promoted the antioxidant activity of starch since the much lower antioxidant activity was determined in native rice starch. Additionally, both DPPH and FRAP results were found to correlate well by total phenolic and flavonoid content. Similar results were previously reported by Dupuis et al. (2017) who aimed to synthesize vanillic acid–amylose complex.

### 3.5 In vitro starch digestibility, eGI, and RS content of modified Rice starch

**FIGURE 2:**
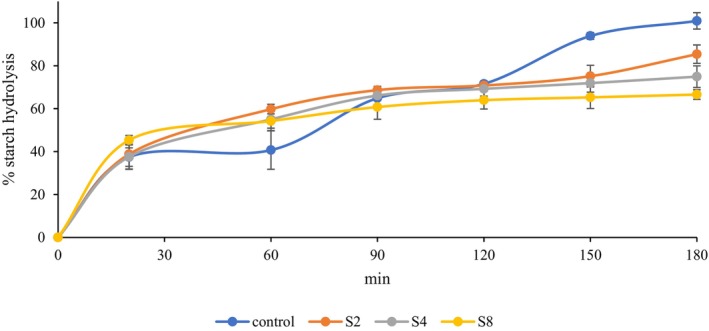
Starch hydrolysis index of matcha extract‐added starch samples. Control, untreated with MTE; S2, treated with 10% MTE (v/w of starch); S4, treated with 20% MTE (v/w of starch); S8, treated with 40% MTE (v/w of starch).

The data indicating in vitro starch hydrolysis rate of ME‐treated rice samples is given in Figure 2. There was a sharp rise and fall in the chart of the control sample, whereas the slope of the ME‐treated samples was more stable. In the first 20 min of digestion, there was a sharp increase in these samples meaning rapidly digestible starch content (RDS). Between 20 and 120 min of the test period, a slow rise was seen in S2, S4, and S8 samples. In this period, starch converted to glucose is named as slowly digestible starch (SDS). In general, the high RDS and the lower SDS content are correlated with higher calorific and glycemic index foods. Thus, in the formulation of diabetic and functional foods, lower SDS and higher RDS content are desired. After 120 min, the starch hydrolysis rate of extract‐treated samples remained nearly stable. At the end of the 180‐min test period, the highest starch hydrolysis was obtained in the control sample, and it was followed by S2, S4, and S8. In the S8 sample containing the highest amount of bioactive compounds, the total starch hydrolysis rate was nearly 65%. The addition of tea extracts can significantly affect the digestibility of starch. Xie et al. (2019) found that the addition of epigallocatechin (EGC) and epigallocatechin‐3‐gallate (EGCG) from tea polyphenols can reduce the digestion extent of wheat starch. The authors attributed this result to strong inhibitory effect of tea polyphenols on amylase enzyme. Similarly, Ayim et al. (2019) reported that tea extracts, particularly those from green tea, can inhibit starch digestibility, with specific compounds such as EGC, and EGCG playing a key role. Fu et al. (2020) determined that tea product utilization was effective in reducing the starch digestibility of cooked rice and matcha had the most significant effect compared to instant green or black tea. Guo et al. (2019) declared that green tea polyphenols decreased the digestibility of lotus seed starch, and the application of high hydrostatic pressure was effective in obtaining these results.

The fitting data of ME‐treated starch obtained by in vitro starch digestion kinetic parameters measured at the different times samples is given in Table 3. The highest AUC was obtained in the control sample as 24,879 and the modification of starch by MTE significantly decreased the AUC level. As a positive correlation, the decrease in AUC also caused a decrease in HI, and the lowest value was obtained for S8 sample as 45.39. AUC and HI were used in the calculation of eGI. The ME treatment was significantly effective in the decrease of eGI of native rice starch and the lowest eGI was determined in the S8 sample as 64.63. The cut‐off value for GI is <55 as low, 55–70 as a medium, and >70 as high GI food (Li et al., 2021). From these results, it can be revealed that ME treatment can be used to produce low GI starch. GI decreasing effect of phenolic compounds may be sourced from the inactivation of amylolytic enzymes or decreasing availability of starch by complexing with amylose or amylopectin. Miao et al. (2015) found that green tea polyphenols especially ECG showed stronger inhibitory activity on human α‐amylase through binding active site residues. It can be concluded that the modification of various starch by ME can be an effective way to decrease the conversion of starch to glucose; thus, the glycemic response of starch can be decreased.

**TABLE 3:** Area under the curve (AUC), hydrolysis index (HI%), expected glycemic index (eGI), and resistant starch (RS) of matcha tea extract‐treated rice starch samples.

Sample	AUC	HI%	eGI	RS
S0	24,879 ± 844^a^	100.00 ± 0.0^a^	94.61 ± 0.00^a^	0.90 ± 0.07^c^
S2	14,135 ± 1018^b^	56.74 ± 2.17^b^	70.86 ± 1.19^b^	14.61 ± 3.03^b^
S4	13,081 ± 911^b^	52.51 ± 1.88^b^	68.54 ± 1.03^b^	25.10 ± 3.55^ba^
S8	11,297 ± 558^b^	45.39 ± 0.70^c^	64.63 ± 0.39^c^	33.43 ± 1.59^a^

*Note*: The superscript letters, in the same column, indicate that are significantly different by Duncan's multiple range test (*p <* .05).

Abbreviations: S0, untreated with MTE; S2, treated with 10% MTE (v/w of starch); S4, treated with 20% MTE (v/w of starch); S8, treated with 40% MTE (v/w of starch).

Research consistently shows that the addition of polyphenols to food can decrease its GI. Thompson et al. (1984) found a negative correlation between polyphenol concentration and glycemic index in leguminous and nonleguminous foods. Coe and Ryan (2016) further supported this by the systematic review, demonstrating that polyphenol‐rich sources can reduce peak and early‐phase glycemic response and maintain the response in later stages of digestion. Fu et al. (2020) determined that the eGI of cooked rice decreased from 77.55 to 66.86 by 3% matcha utilization and attributed these results to modified structure and crystal type of starch (from binding to tea products) affected the action site of the enzyme. These findings collectively suggest that polyphenol addition can indeed lower the glycemic index of food.

RS is classified as an undigested starch fraction after 180 min in vitro starch digestion. According to Table 2, ME treatment had a significant effect on the RS content of rice starch. The highest RS content was determined in the S8 sample as 33.43%, and it was followed by 25.10% and 14.61% in S4 and S2 starch samples, respectively. The lowest RS content was also obtained in native rice starch by 0.90%. It can be considered that by 40% (v/w of starch) ME addition in gelatinized rice starch, RS content could be increased nearly 30 times higher. Different two factors may be effective in the increment of RS. First of all, phenolics of the extract may inhibit starch degradation enzymes and after 180 min, a high content of starch remained undegraded to glucose. Additionally, the second reason may be that a complexation reaction may have occurred between starch and tea polyphenols, and thus starch is protected from digestion. Hernández et al. (2022) explained that the content of resistant starch can be increased by forming starch–polyphenol V‐type complexes. It was also considered that the RS content was significantly higher when shorter chain‐length polyphenols were used (Gutierrez et al., 2020). On the contrary, Barros et al. (2014) found that higher molecular weight polymeric proanthocyanins from sorghum were more effective in increasing RS formation. Li et al. (2021) aimed to use matcha tea in the formulation of rice noddle to decrease the glycemic response of the final product. The authors found that the utilization of matcha tea significantly increased the RS content of noodles from 7.56% to 25.94%, and the possible reason was explained as well as our previous discussions. Fu et al. (2020) found that the RS content of cooked rice increased from 17.06% to 25.04% by 3% matcha utilization.

## 4 CONCLUSION

In recent years, the market for health‐promoting foods has grown, with a focus on combating the prevalence of chronic diseases. Native starch is the main carbohydrate source, and the majority of daily energy needs are met by starch. Conversely, starch lacks the most bioactive compound. By modification of starch with phenolic‐rich extracts, the poor nutritive value of starch can be improved. According to the study, it was determined that the treatment of rice starch with matcha extract was effective in decreasing of eGI of rice starch. In addition, at the highest level of ME addition (S8), the RS content was increased almost 30‐fold. Moreover, the total phenolic, flavonoid and antioxidant content of native rice starch were enhanced by ME treatment. The results of the study have several important implications for the modification of starch by various tea compounds to increase its nutritional value. Moreover, further studies should be carried out to determine the effect of different phytochemicals on starch digestion.

## AUTHOR CONTRIBUTIONS

**Hümeyra Cetin‐Babaoglu:** Data curation (equal); formal analysis (equal); investigation (equal); methodology (equal). **Hümeyra Aydın:** Formal analysis (equal); investigation (equal); project administration (equal). **Rumeysa Kumas:** Formal analysis (equal). **Sultan Arslan‐Tontul:** Conceptualization (equal); methodology (equal); resources (equal); supervision (equal); writing – original draft (equal); writing – review and editing (equal).

## CONFLICT OF INTEREST STATEMENT

The authors declare that they have no conflicts of interest.

## Data Availability

The data that support the findings of this study are available on request from the corresponding author.
